# Feature Selection for Object-Based Classification of High-Resolution Remote Sensing Images Based on the Combination of a Genetic Algorithm and Tabu Search

**DOI:** 10.1155/2018/6595792

**Published:** 2018-01-18

**Authors:** Lei Shi, Youchuan Wan, Xianjun Gao, Mingwei Wang

**Affiliations:** ^1^School of Remote Sensing and Information Engineering, Wuhan University, Wuhan 430072, China; ^2^School of Geoscience, Yangtze University, Wuhan 430100, China

## Abstract

In object-based image analysis of high-resolution images, the number of features can reach hundreds, so it is necessary to perform feature reduction prior to classification. In this paper, a feature selection method based on the combination of a genetic algorithm (GA) and tabu search (TS) is presented. The proposed GATS method aims to reduce the premature convergence of the GA by the use of TS. A prematurity index is first defined to judge the convergence situation during the search. When premature convergence does take place, an improved mutation operator is executed, in which TS is performed on individuals with higher fitness values. As for the other individuals with lower fitness values, mutation with a higher probability is carried out. Experiments using the proposed GATS feature selection method and three other methods, a standard GA, the multistart TS method, and ReliefF, were conducted on WorldView-2 and QuickBird images. The experimental results showed that the proposed method outperforms the other methods in terms of the final classification accuracy.

## 1. Introduction

With the development of satellite remote sensing technologies, more and more high spatial resolution images are now becoming available. High spatial resolution images have been widely and successfully utilized in land-cover classification [1]. As smaller-scale ground objects can be identified and more detailed information can be obtained from high-resolution images, the traditional pixel-based image analysis methods cannot satisfy the classification demands of high-resolution remote sensing images, because of the low accuracy and the insufficient utilization of the rich information [2]. In object-based classification approaches, by grouping pixels together with a specific method, the images are segmented into homogeneous regions named “objects,” which can provide not only spectral information, but also texture features, shape features, and neighboring relationships for the classification [3]. Therefore, in high-resolution image classification, it is reasonable to use object-based methods instead of pixel-based methods. A large number of studies have compared pixel-based and object-based classification techniques, and it can be concluded that the classification accuracies obtained by the object-based methods are higher than those obtained by the pixel-based methods [4].

The dimensions of the features extracted from image objects are much larger than pixels, which mainly contain spectral-based information (e.g., mean, ratio, and standard deviation) [5]. In object-based classification, hundreds of features involving the spectral, geometry, and texture features can be obtained from the image objects. However, large amounts of features participating in classification always give rise to the “dimension disaster,” which decreases the classification accuracy. As some features make contributions to the classification and others have less influence on the result, features are commonly divided into relevant features, redundant features, and irrelevant features [2]. To yield better classification results, the irrelevant information should be removed, as much as possible, and the utilization of relevant information should be maximized. Therefore, feature selection prior to the object-based classification of high-resolution remote sensing images is a prerequisite. After the redundant and irrelevant features are removed, the training time is reduced and the classification efficiency can be improved [6].

The task of feature selection is to obtain the optimal feature subset to achieve a similar or better classification quality than when using all the features [6]. Various approaches have been put forward for feature selection, including the branch and bound method [7], the sequential forward selection (SFS) method [8], the sequential backward selection (SBS) method [9], and ReliefF [10]. However, a variety of problems still exist in the above methods, including the high computational complexity, monotonicity of the objective function, and insufficient consideration of the correlations between features. The feature selection process is actually a kind of combinatorial optimization problem; therefore, intelligent search algorithms can be used to solve the problem. Evolutionary computation (EC) methods have been applied in feature selection problem and achieved much success recently [11]. For example, as a global optimization method developed from the genetic process of natural selection, genetic algorithms (GA) have been widely used in feature selection studies [12–14] because of their robustness and fast searching speed. With mutual information as the evaluation function, Huang et al. [15] searched for the optimal feature subset using a GA, with consideration of the correlations not only between candidate features and classes, but also between candidate features and selected features. Yan et al. [16] carried out feature selection using an adaptive GA, in which the probabilities of crossover and mutation for each individual depend on their fitness values. In this way, superior individuals can be found and the convergence speed is increased. However, in terms of population diversity, there is still room for improvement. Therefore, further study and improvement of the use of GAs for feature selection are necessary.

Tabu search (TS) is also a typical way to solve optimization problems [17], and it has been performed successfully in the combinatorial optimization field. Danenas et al. [18] developed a credit risk evaluation method based on TS and the correlation-based feature subset evaluator. Based on TS and variable neighborhood search, Sicilia et al. [19] presented an optimization algorithm to solve the problem of vehicle routing in urban areas. Moreover, combinations of TS and other approaches have been proved to be able to solve different optimization problems. For example, by combining the advantages of simulated annealing (SA) and TS, Katsigiannis et al. [20] presented a hybrid method named SA-TS to solve the optimal sizing problem of autonomous power systems. Shen et al. [21] proposed a gene selection method based on a combination of particle swarm optimization (PSO) and TS for tumor classification. A continuous tabu simplex search (CTSS) method was developed by Chelouah and Siarry [22] by the combination of TS and the Nelder-Mead simplex approach to solve global optimization problems in multiminima functions.

In El Ferchichi et al.'s work [23], both GA and TS were used to select optimal feature subsets based on data from transport system and they found that each method had its own advantages in terms of processing speed or dimensionality reduction. Through an investigation of the performance of the GA and TS, it is not difficult to see that the weakness of TS is the dependence on the initial solutions and the slow speed of convergence, while the problem with the GA is premature convergence. However, in theory, the GA could provide good initial solutions for TS and, in return, the characteristics of TS could help the GA to escape from premature convergence. Consequently, a novel feature selection method named GATS based on the combination of a GA and TS is proposed in this paper.

The rest of this paper is organized as follows. In Section 2, the main principle of the proposed GATS method is introduced. The implementation of the GATS method is detailed in Section 3. The experimental results and discussions are provided in Section 4. Finally, the conclusions are made in Section 5.

## 2. Introduction to GATS

### 2.1. Overview of the Genetic Algorithm (GA)

**Figure 1:**
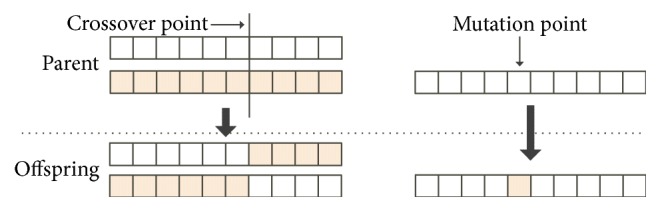
Crossover and mutation operators of the genetic algorithm.

As a random heuristic search algorithm inspired by natural evolutionary laws, the GA was first proposed by Holland in 1975 [24]. To solve a problem by the use of the GA, the first step is to establish the initial population. Each member of the initial population is called an “individual” (or chromosome), corresponding to a solution to a certain problem [19]. Commonly, fitness is used to represent a chromosome's adaptability to the environment, so each chromosome is evaluated by a certain objective function [25]. A selection operation is then carried out as it picks the individuals with higher fitness values, which are used to regenerate new offspring [26, 27]. After this, crossover is an essential step to produce new individuals by randomly recombining the selected parent chromosomes on a random crossover point with a specific probability. Finally, a mutation operation is implemented with a relatively small probability, which can reduce the appearance of local optima by randomly replacing one or more genes of the current chromosomes [13, 28]. The crossover and mutation operators of the GA are illustrated in Figure 1.

### 2.2. Overview of Tabu Search (TS)

As a metasearch strategy first put forward by Glover [17, 29], the TS algorithm has been widely used to solve combinatorial optimization problems. By starting with an initial feasible solution *X*, TS conducts the search in the neighborhood solutions of *X* generated by neighborhood moves (explained later). The value of the best solution so far *X*_best_ is initially assigned the value of *X*. Supposing that *X*′ is the best among the neighborhood solutions, then the value of *X* is replaced with the value of *X*′ in two cases: *X*′ is not included in the tabu list (explained later); and *X*′ is included in the tabu list, but it satisfies the aspiration criterion (explained later). At the same time, if the new solution *X*′ is superior to *X*_best_, the value of *X*_best_ is replaced with the value of *X*′. The move from *X* to *X*′ is then recorded by the tabu list, which means that this move is forbidden in a certain number of iterations. The neighborhood search is continued based on the new feasible solution *X*′. This whole procedure is iteratively executed until the stopping condition is satisfied. After the iterative process has ended, the current best solution so far *X*_best_ is the final optimal solution provided by the TS method [30].

**Figure 2:**
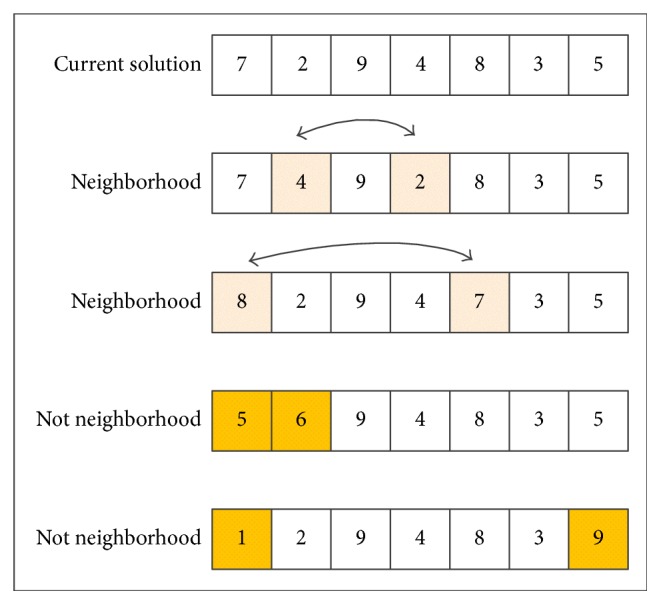
Neighborhood moves of GATS.

In this section, we explain the key elements in the TS method, as mentioned above: the neighborhood moves, the tabu list, and the aspiration criterion. Commonly, neighborhood moves can be realized in several ways, for example, increasing or decreasing the values of the chromosome genes by one and reversing the positions of two genes belonging to the same chromosome. In this paper, the position exchange pattern is adopted. As shown in Figure 2, supposing that one current solution *X* can be expressed by {7, 2, 9, 4, 8, 3, 5}, it is not difficult to judge that $\left\{7,\underline{4},9,\underline{2},8,3,5\right\}$ and $\left\{\underline{8},2,9,4,\underline{7},3,5\right\}$ belong to the neighborhood of *X* resulting from position exchange of the underlined elements. Meanwhile, $\left\{\underline{5},\underline{6},9,4,8,3,5\right\}$ and $\left\{\underline{1},2,9,4,8,3,\underline{9}\right\}$ are not neighborhood moves of *X* because the values of the underlined elements have been changed. The tabu list is a kind of short-term memory table and is used to deposit the latest neighborhood moves which are forbidden for *L*_*t*_ (length of the tabu list) times. In this way, local optima can be effectively avoided. Generally, the first input first output (FIFO) strategy is used to deal with the updating of elements in the tabu list, which means that, after *L*_*t*_ times iterations, the element is released from the tabu list and the tabu property for this move is removed. However, once a move included in the tabu list leads to a better solution than *X*_best_, the tabu property is ignored because it led the searching to obtain the best solution so far. Accordingly, its solution replaces the current solution *X* and the best solution so far *X*_best_. This is the so-called aspiration criterion. On the one hand, it can help to prevent the loss of superior solutions during the iterations; on the other hand, it can encourage the movement to unexpected solution fields to further realize the global search [31].

### 2.3. Basic Principle of the GATS Method

As previously mentioned, premature convergence is the main problem of the GA, and the weaknesses of TS is its dependence on the initial solutions and the single-point search mode. Fortunately, the GA can provide high-quality initial solutions for TS, and its fast searching speed can compensate for the speed problem of TS. Moreover, the flexible tabu list and the aspiration criterion of TS can help the GA to escape from local optima. In the proposed GATS method, TS is integrated with the GA in the following way: the mutation operator based on TS replaces the original mutation operator once the prematurity warning has been triggered. Evolutionary computation approaches refined by local search methods could be termed as memetic algorithms which have been successfully applied in many studies [32–35]. And a memetic framework has also been utilized in the proposed feature selection method.

To judge whether or not the search process has been trapped in premature convergence, the prematurity index is defined through calculating the similarity degree between each two individuals as follows:

(1) $$\begin{array}{c} I=\overset{N-1}{\underset{i=1}{\sum }}\overset{N}{\underset{j=i+1}{\sum }}\frac{\left(N-2\right)!*2!}{N!}*\frac{W}{L}, \end{array}$$

where *I* is the prematurity index, *N* is the count of individuals of the population, *N*!/((*N* − 2)!*∗*2!) is the count of the combinations of pairwise individuals from the population, *W* is the count of the same genes with the same locations for each two individuals among the whole population, and *L* is the length of the chromosome.

During each iteration of the feature selection procedure, the prematurity index is calculated after the crossover step to judge if the prematurity problem has occurred. Once prematurity does happen, all the individuals in the current population are first sorted in descending order by values of fitness. The TS is then performed based on the top 50% of the sorted individuals, and the mutation operator is executed on the others with a high probability. By the use of the proposed GATS method, on the one hand, the prematurity problem of the GA is improved and, on the other hand, TS can start searching with a batch of favorable initial solutions instead of a common one. In this way, both the GA and TS can give full play to their respective advantages in the optimization search problem.

## 3. The Proposed GATS Methodology

### 3.1. Coding Scheme

**Figure 3:**
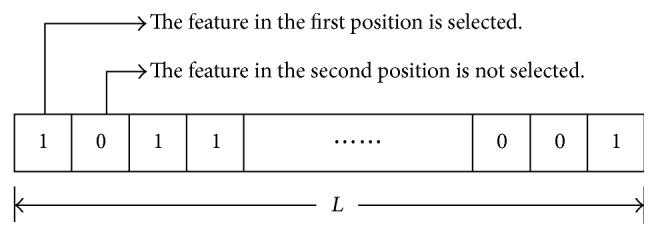
Binary encoding scheme.

The binary coding scheme is the most commonly used coding technique, and its encoding and decoding are simple. It is also easy to realize genetic operators, including crossover and mutation, in the binary coding scheme. Therefore, the binary coding method is adopted in this paper to express the chromosomes in the GATS feature selection procedure. As shown in Figure 3, we suppose that the number of all the candidate features is *L*, then the length of each chromosome is *L*, and each gene of the chromosome corresponds to one feature. When a gene from one chromosome is expressed as “1,” it means that the corresponding feature has been selected; when the gene is marked as “0,” it means that this feature has not been selected.

### 3.2. Objective Function

The objective function in the GA is designed to calculate the fitness values, which can be used to evaluate the viability of the individuals. A set of good features can separate classes quite precisely by making the within-class distance as short as possible and the between-class distance as long as possible. In this paper, the within-class and between-class distances are chosen as the main factors to form the objective function. And they are calculated as follows:

(2) Dw=∑i=1C∑j=1niXij−ViTXij−Vin,Db=∑i=1C∑j=i+1CVi−VjTVi−VjNC,

where *D*_*w*_ is the within-class distance, *C* is the count of all the classes, *n*_*i*_ is the count of the samples from class *i*,  *X*_*ij*_ is the feature vector of sample *j* from class *i*, *V*_*i*_ is the feature vector of the center belonging to class *i*, and *n* is the count of all the samples. *D*_*b*_ is the between-class distance, and *N*_*C*_ is the count of the combinations between classes which can be calculated by *C*!/(*C* − 2)!2!. Based on the principle of minimizing the within-class distance and maximizing the between-class distance, the objective function can finally be expressed as follows:

(3) $$\begin{array}{c} F=\frac{D_{b}}{D_{w}+d}, \end{array}$$

where *F* is the fitness value and *d* is an extremely small constant (here, *d* = 10^−10^) in the case that the value of *D*_*w*_ is zero.

### 3.3. Procedure of GATS

**Figure 4:**
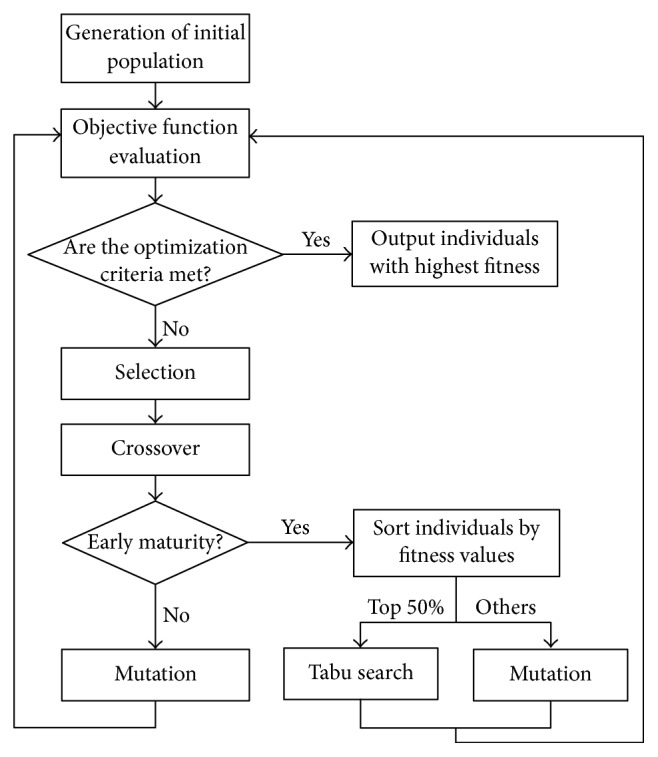
Flowchart of the GATS method.

A flowchart of the proposed GATS method is shown in Figure 4. The implementation of the whole procedure can be explained as follows.

**Step 1 (initial population).**

Individuals with the length of *L* are randomly generated to form the initial population with the size of *N*.

**Step 2 (objective function).**

Values of fitness for each individual are calculated by (3).

**Step 3 (selection).**

The purpose of selection is to retain the superior individuals with higher fitness values. As a classical random selection technology, roulette wheel selection [36] (also named proportional selection) is adopted in GATS. In the roulette wheel selection method, the selected probability of each individual is proportional to the value of its fitness. When the size of the population is *N*, the chosen probability for one individual can be calculated as follows:

(4) $$\begin{array}{c} P_{i}=\frac{F_{i}}{\sum_{i=1}^{N}F_{i}}, \end{array}$$

where the probability of being chosen for individual *i* is *P*_*i*_, and the fitness value of individual *i* is *F*_*i*_. Individuals with higher fitness values are more likely to be selected.

**Step 4 (crossover).**

By exchanging the genes of two parent individuals with a certain crossover probability *P*_*c*_, as shown in Figure 1, two offspring individuals are produced. Through the crossover operation, the information of the individuals is sufficiently recombined and the search range is effectively expanded.

**Step 5 (judgment of prematurity).**

As the proposed GATS method is used to improve the premature convergence problem of the GA, the detection of prematurity is quite important. The prematurity index *I* is calculated by (1). Through a large number of experiments, the threshold value is derived, and once the value of *I* is larger than threshold *T*_*p*_, Step 8 is executed. Otherwise, it means prematurity has not yet occurred, so go to Step 7.

**Step 6 (conventional mutation).**

As an important operation, this step simulates gene mutation of the biological evolution process. The mutation operation is executed on a random gene of parent individuals by changing it from “1” to “0” or from “0” to “1” with a specific mutation probability *P*_*m*_. Then go back to Step 2.

**Step 7 (the improved mutation).**

This is the key step in helping the search procedure to jump out of the local convergence situation. When premature convergence occurs, TS is carried out based on the superior individuals with higher fitness values. The conventional mutation operation with a higher probability *P*_*mt*_ is performed on individuals with lower fitness values. The proportion of superior individuals is set to 50% in GATS. Then go back to Step 2.

**Step 8 (termination condition).**

In GATS, after a specified number of iterations, the feature selection process stops.

It is worth mentioning that voting technology is utilized in GATS to obtain optimal feature subsets with a quantitative size, as the count of features selected by the proposed method is uncertain. At first, the above procedures are carried out iteratively for a certain number of times (50 times in this paper). Statistical analysis is then conducted based on the above feature selection results, and the features are ranked according to the number of times they are selected. Finally, the features with the highest number of selections are included in the optimal feature subset.

## 4. Results and Discussion

The proposed GATS method was realized by visual C++ programming language on a computer with a 3.10 GHz CPU and 4.00 GB RAM under the Windows 7 operating system.

### 4.1. Experimental Design

**Figure 5:**
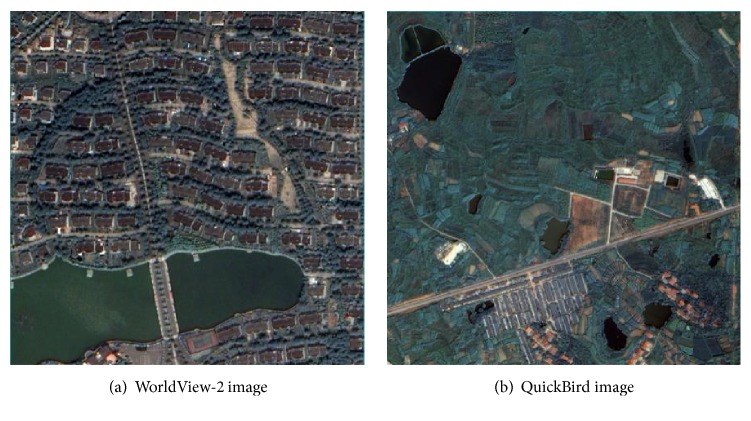
Experimental images.

As shown in Figure 5, a WorldView-2 image with a 0.5 m spatial resolution and a QuickBird image with a 0.6 m resolution were, respectively, used in two experiments to verify the proposed method. The experimental regions are both located in the city of Wuhan, Central China. As shown in Figure 5(a), the first study site is a typical urban area, with the land-cover types including buildings, vegetation, water, shadows, and ground surfaces. The second study area displayed in Figure 5(b) is a complex suburban area, with vegetation, water, buildings, ground surfaces, bare land, and secondary bare land.

**Figure 6:**
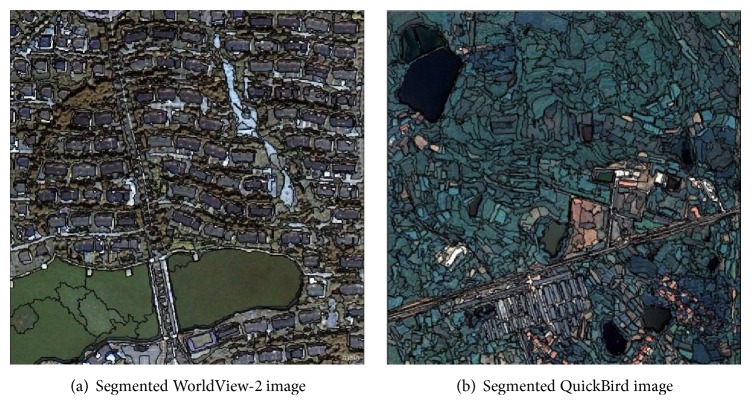
Segmented images.

As the first step of the whole classification procedure, a bottom-up region merging method is employed in GATS to segment the images. Through segmentation experiments, the settings of the parameters were decided and are shown in Table 1.  Figure 6 shows the final segmentation results of the WorldView-2 and QuickBird images. After the segmentation process, 790 objects for the WorldView-2 image and 1319 objects for the QuickBird image were finally obtained, and 249 features were extracted from the objects, as shown in Table 2. All the texture features listed in the table were derived from the gray-level cooccurrence matrix (GLCM) proposed by Haralick et al. [37]. And then, 77 training samples for the WorldView-2 image and 96 training samples for the QuickBird image were randomly selected by manual work for both the feature selection and subsequent classification procedure.

Based on the above analysis and preprocessing of the experimental images, the GATS method was executed on the 249 features to select the optimal feature subset.  Table 3 shows the details of the parameter settings for the GATS feature selection method and as parameters of GA and TS are commonly used, most of these parameters are assigned values empirically according to the practical problems [26, 38, 39]. As the main purpose of GATS is to improve the premature convergence of the GA, a comparison between GATS and the GA is essential to confirm the effectiveness of the proposed method. To prove that the GA can provide multiple initial solutions with a high quality for TS, a multi-initial-solution TS method with common initial solutions was utilized for the comparison. In addition, as a typical feature selection technology, ReliefF [2] was also compared with GATS. In summary, three experiments based on standard GA, a multistart TS approach [40], and the classical ReliefF algorithm were carried out in this study. In the experiments, the values of the parameters for the GA and TS were kept the same as GATS. In the experiment with ReliefF, the sampling times *S*_*r*_ were 500 and 800, the number of neighbor points *N*_*r*_ was 5 and 10, and the threshold values of the feature weight *T*_*R*_ were 0.8 and 0.8 for the WorldView-2 and QuickBird images, respectively.

### 4.2. Experimental Results

Table 4 lists the numbers of selected features, the mean and standard deviation of both fitness values and CPU time of GATS, GA, multistart TS, and ReliefF methods through 50 times runs. As a typical highly efficient feature weighting method, the time taken by the ReliefF algorithm is shorter than the other three methods. Among the other three methods, although the GA costs the least time, the number of features obtained by this method is always the largest, and its mean fitness values are the lowest. In the multistart TS method, as several initial solutions are used in the search instead of a single one, the CPU time is longer than for the GA, but the fitness values are higher. As for the proposed GATS method, the time required is longer than for the multistart TS method, but the feature extraction effect is much better as the number of optimal features obtained by GATS is much smaller. Most importantly, the mean values of fitness obtained by the proposed GATS method are obviously improved compared with the other methods. In addition, statistical analysis has been conducted based on above experiments and standard deviation values for fitness and CPU time have been obtained. It is not hard to see that both items get low values which demonstrate the high stability and reliability of the proposed method.

Table 5 lists the final feature selection results of each method (the number of features participating in the following classification was uniformly set to seven for all the experiments). It is not difficult to observe that, as important information in object-based high-resolution image analysis, texture features such as GLCM mean are always selected by the proposed method, but, for the other methods, texture is ignored in most cases, and the spectral information occupies the dominant position.

**Figure 7:**
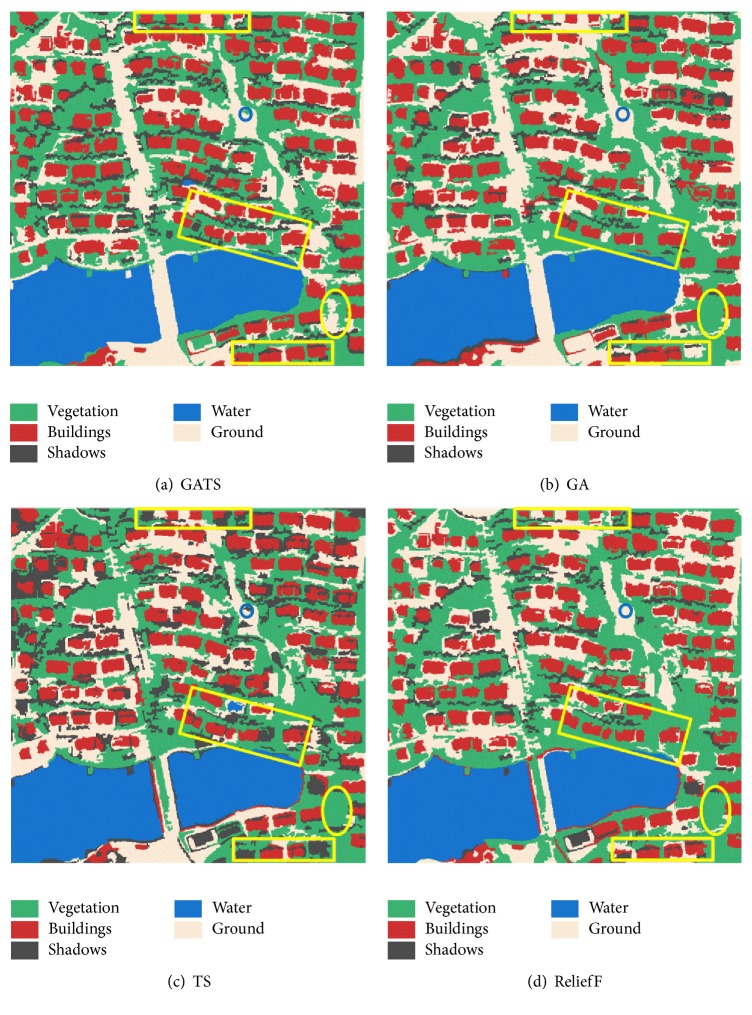
Classification results for the WorldView-2 image.

**Figure 8:**
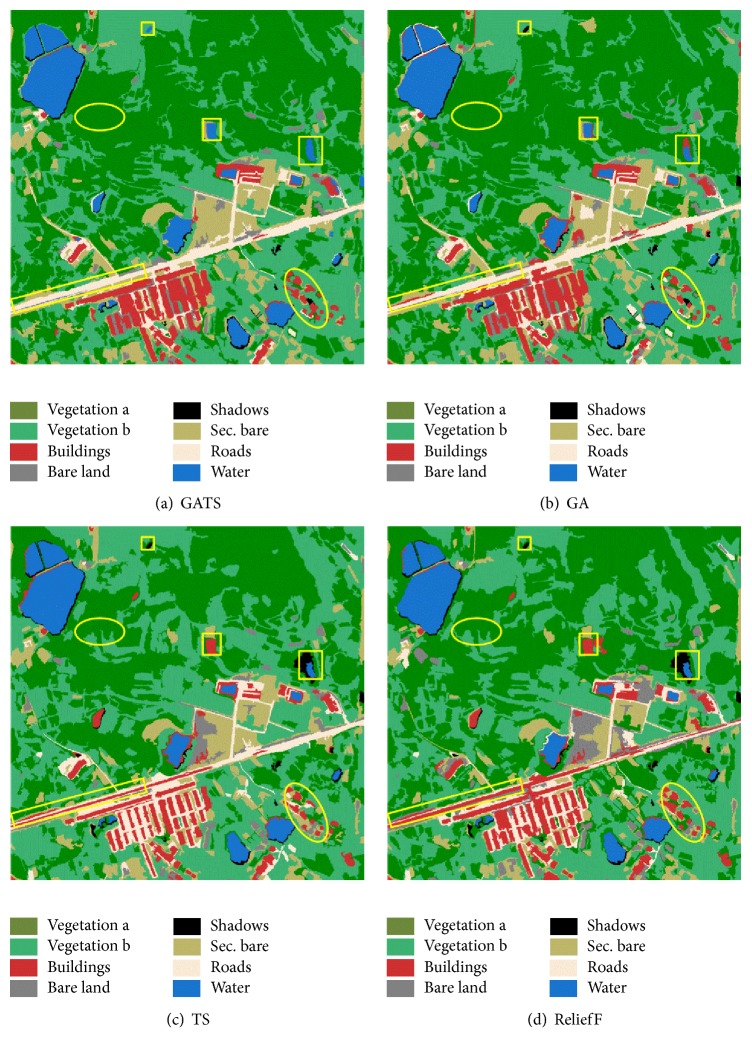
Classification results for the QuickBird image.

As one of the most effective machine learning algorithms, support vector machine (SVM) has been widely employed in the classification of remote sensing images [41–44]. Using the above feature selection results, SVM was adopted to classify the WorldView-2 and QuickBird images. Through calculation of the *k*-fold cross-validation method, the values of C and Gamma (the parameters of SVM) were, respectively, set to 100 and 5 for the WorldView-2 image and 32 and 0.5 for the QuickBird image. The final classification results are shown in Figures 7 and 8.

For the WorldView-2 classification results, it can be observed that buildings with more accurate contours and higher integrity are provided by the GATS method, as highlighted with the yellow rectangle in Figure 7(a). Meanwhile, the extraction of buildings by GA and ReliefF is incomplete, and, for TS, some of the buildings are misclassified as water and shadows. In terms of a place featuring a mixture of ground and vegetation, as highlighted by the yellow ellipse, it can be distinguished by GATS, whereas the other methods result in misclassification. As highlighted by the blue circle, the small area of vegetation surrounded by other large-scale objects can also be successfully recognized by the proposed method. The TS method misclassifies the small vegetation object as shadow, and GA and ReliefF fail to recognize it.

As shown in Figure 8, for the QuickBird image, there are several miss-extractions of buildings in the results of the other three methods, as highlighted by the yellow ellipse in the bottom right corner of the image. The extraction of the main road in Figure 8(a) by GATS is more complete than for the other methods. Despite the high similarity of the spectral characteristics, the water, buildings, and shadows in Figure 8(a) are less likely to be misclassified because of the participation of the texture information in the classification. From the visual assessment of the classification results, it is not difficult to conclude that, in general, the proposed method leads to a preferable classification effect.

### 4.3. Accuracy Analysis and Discussion

As the final objective of analyzing images by the proposed GATS method is to improve the classification accuracy, confusion matrices are used to quantitatively evaluate the performance of the different methods. Producer's accuracy, user's accuracy, overall accuracy, and Kappa coefficient calculated from the matrix are the key indicators to assess the classification quality in Figures 7 and 8.

The producer's accuracy refers to the probability of a reference sample being correctly classified [45]. It can be calculated by

(5) $$\begin{array}{c} PA=\frac{x_{ij}}{\sum x_{+j}}*100\%, \end{array}$$

where PA represents the producer's accuracy, *x*_*ij*_ refers to the element in the *i*th row and *j*th column of the matrix, and ∑*x*_+*j*_ represents the sum of the elements from the *j*th column of the confusion matrix.

The user's accuracy represents the probability of the classified land being grouped into the true ground reference category:

(6) $$\begin{array}{c} UA=\frac{x_{ij}}{\sum x_{i+}}*100\%, \end{array}$$

where UA represents the user's accuracy and ∑*x*_*i*+_ is the sum of the elements from the *i*th row.

The overall accuracy refers to the percentage of correctly classified samples and can be calculated by

(7) $$\begin{array}{c} OA=\frac{\sum_{i=1}^{C}x_{ii}}{\sum_{i=1}^{C}\sum_{j=1}^{C}x_{ij}}*100\%, \end{array}$$

where OA represents the overall accuracy, *C* is the dimension of the confusion matrix (also the number of classes), and *x*_*ii*_ is the sum of the elements in the confusion matrix's diagonal.

To measure the classification ability of the utilized method with respect to a random classification, the Kappa coefficient is obtained by statistical calculation of the confusion matrix and can be expressed as follows:

(8) $$\begin{array}{c} K=\frac{n*\sum_{i=1}^{C}x_{ii}-\sum \left(x_{i+}*x_{+j}\right)}{n^{2}-\sum \left(x_{i+}*x_{+j}\right)}, \end{array}$$

where *K* represents the Kappa coefficient and *n* is the total number of samples. Generally speaking, the higher the above accuracies or coefficients, the better the classification effect.

Overall accuracies and Kappa coefficients for both images are listed in Table 6. For the WorldView-2 image, the GATS method produces a significantly better classification effect than the other methods, with increments of at least 11.5% in OA and 0.14 in Kappa. In terms of the QuickBird image, the highest OA of 88.25% is yielded by GATS.

Table 7 lists the producer's accuracies and user's accuracies for each class of the WorldView-2 image. Due to the spectral similarity between buildings and ground, the lowest producer's accuracy of 75.81% for buildings and the lowest user's accuracy of 52.08% for ground are yielded by the TS and GA methods, respectively. However, with the assistance of the texture information provided by the GATS feature selection method, both the producer's and user's accuracies of buildings reach 91.94%, and the accuracy of ground is also much higher than for the other methods. The above results indicate that texture features can play a key role in differentiating classes with similar spectral characteristics. Although ReliefF outperforms the other methods in CPU time, it provides the lowest user's accuracies of 51.95% and 47.5%, respectively, for vegetation and shadows.

The producer's accuracies and user's accuracies for the QuickBird image are listed in Table 8. Compared to the other classes, the buildings class shows the biggest increase in user's accuracy with the GATS method. Both the producer's and user's accuracies for vegetation 1 and vegetation 2 provided by GATS reach a stable level of 90%, whereas the accuracies yielded by the other methods are mainly between 70% and 80%. Not only does ReliefF obtain the lowest overall accuracy and Kappa, but it also yields the lowest user's accuracy of only 50% for bare land and the lowest producer's accuracy of 55% for roads.

## 5. Conclusions

In this paper, we have put forward a feature selection method based on the integration of GA and TS (GATS). The proposed GATS method is aimed at improving the premature convergence of the GA with the new mutation operator modified by TS. To validate the reliability and effectiveness of the proposed feature selection method, other feature selection methods, a traditional GA, multistart TS, and ReliefF, were also implemented. SVM was then utilized to classify the WorldView-2 and QuickBird images based on the selected features. Through the experiments and comparisons, it was demonstrated that the proposed GATS method can increase the classification accuracy by providing feature subsets with the within-class distances as small as possible and the between-class distances as big as possible. However, the proposed method could be further improved in terms of feature number. As restriction of the feature number is not easy to implement in the binary coding scheme, voting technology is used in GATS to select a fixed number of features from the feature selection results. Therefore, in our future work, a novel coding scheme for GATS will be studied, by which control of the optimal feature number will be realized.

## Figures and Tables

**Table 1:** Segmentation parameters.

Image	Parameter		
	Scale	Shape	Compactness
WorldView-2	48	0.1	0.6
QuickBird	80	0.2	0.6

**Table 2:** List of object features.

Feature category	Object features	Number of features
Spectral	Mean, Brightness, NDVI, NDWI, HSI, Ratio, Standard deviation, Skewness, etc.	22
Geometry	Length/Width, Shape index, Area, Volume, Compactness, Density, Asymmetry, etc.	27
Texture	Homogeneity, Contrast, Dissimilarity, Entropy, Mean, StdDev, Correlation, Ang. 2nd moment, etc.	200

**Table 3:** Parameter settings of GATS.

Parameter	Explanation	Value	
		WorldView-2	QuickBird
*S*	Iterations of the GA	75	100
*N*	Size of the initial population	25	40
*L*	Length of each individual	60	60
*P*_*c*_	Crossover probability	0.8	0.8
*P*_*m*_	Standard mutation probability	0.1	0.1
*P*_*mt*_	Modified mutation probability	0.8	0.8
*I*	Index of prematurity	0.8	0.85
*S*_*t*_	Iterations of TS	40	23
*N*_*t*_	Size of the TS neighborhood	25	10
*L*_*t*_	Length of the tabu list	10	12

**Table 4:** Number of features and CPU time.

	GATS	GA	TS	ReliefF
WorldView-2				
Feature number	45	123	107	86
Mean fitness	12.09	6.25	8.97	—
CPU time (seconds)	31.92	5.90	27	1.93
Std fitness	0.79	0.96	1.09	—
Std CPU time	2.45	1.35	1.96	0.21
QuickBird				
Feature number	67	130	113	99
Mean fitness	17.93	11.63	13.52	—
CPU time (seconds)	37.14	16	32.83	2.37
Std fitness	1.14	1.46	1.63	—
Std CPU time	2.79	1.52	2.46	0.36

**Table 5:** List of features selected by each method.

Data	Method	Features		
		Spectral	Texture	Geometry
WorldView-2	GATS	Mean layer 1/4, Brightness,	GLCM mean layer 3 (0°),	—
		Ratio layer 1, Intensity	GLCM ang. 2nd moment (135°)	
	GA	NDWI, NDVI,	GLCM homogeneity layer 1 (45°),	—
		Ratio layer 1/2/4	GLCM homogeneity layer 3 (45°)	
	TS	NDWI, NDVI,	—	—
		Mean layer 2/4,		
		Ratio layer 1/2/4		
	ReliefF	NDWI, NDVI,	—	Elliptic Fit
		Mean layer 4, Brightness,		
		Ratio layer 1/4		

QuickBird	GATS	NDWI, NDVI,	GLCM ang. 2nd all dir.,	—
		Mean layer 2,	GLCM ang. 2nd moment	
		Ratio layer 1, Intensity	layer 1 (45°)	
	GA	NDWI, NDVI,	—	—
		Mean layer 3/4,		
		Ratio layer 2/3, Saturation		
	TS	NDWI, NDVI,	—	—
		Brightness,		
		Ratio layer 1/2/3/4		
	ReliefF	NDWI, NDVI,	—	—
		Mean layer 3, Saturation,		
		Ratio layer 3/4, Hue		

**Table 6:** Overall accuracy and Kappa for the WorldView-2 and QuickBird images.

	WorldView-2				QuickBird			
	GATS	GA	TS	ReliefF	GATS	GA	TS	ReliefF
OA (%)	89.50	75.50	73.50	78.00	88.25	83.00	74.75	73.25
Kappa	0.86	0.68	0.66	0.72	0.84	0.76	0.65	0.63

**Table 7:** Classification accuracies for the WorldView-2 image.

Class	GATS		GA		TS		ReliefF	
	PA (%)	UA (%)	PA (%)	UA (%)	PA (%)	UA (%)	PA (%)	UA (%)
Buildings	91.94	91.94	85.00	87.93	75.81	89.00	85.48	88.33
Ground	91.43	78.05	71.43	52.08	60.00	52.50	65.71	54.76
Vegetation	90.91	90.91	90.91	83.33	90.91	90.91	81.82	51.95
Water	84.00	93.33	74.00	72.55	68.00	80.95	78.00	79.59
Shadows	90.48	92.68	63.64	90.32	83.33	61.40	76.19	47.50

**Table 8:** Classification accuracies for the QuickBird image.

Class	GATS		GA		TS		ReliefF	
	PA (%)	UA (%)	PA (%)	UA (%)	PA (%)	UA (%)	PA (%)	UA (%)
Buildings	86.67	92.86	80.00	72.73	76.67	69.70	83.33	62.50
Vegetation 1	91.72	88.08	86.21	85.62	66.90	76.98	68.97	76.92
Vegetation 2	86.67	90.91	83.33	85.03	80.00	72.73	79.33	73.46
Water	86.67	86.67	73.33	84.62	66.67	90.91	66.67	90.91
Bare land	84.62	78.42	80.00	66.67	73.33	68.75	66.67	50.00
Sec. bare land	86.67	86.67	86.67	81.25	86.67	76.47	66.67	76.92
Roads	90.00	85.71	80.00	84.21	80.00	80.00	55.00	84.62
Shadows	80.00	87.50	60.00	75.00	90.00	75.00	80.00	72.73
